# Lower Limb Malrotation following Minimally Invasive Plating in Distal Tibia Fractures

**DOI:** 10.5704/MOJ.2403.018

**Published:** 2024-03

**Authors:** WMQ Yap, JW Ng, MJJR Lee, EBK Kwek

**Affiliations:** 1 Department of Orthopaedic Surgery, Tan Tock Seng Hospital, Singapore; 2 Department of Orthopaedic Surgery, Woodlands Health, Singapore

**Keywords:** distal tibia fracture, MIPO, minimally invasive, percutaneous, open reduction and internal fixation

## Abstract

**Introduction:**

Minimally invasive percutaneousosteosynthesis (MIPO) plating techniques havedemonstrated good outcomes in the treatment of distal tibia fractures. Early arthritis and functional impairment mayoccur if length and rotation are not restored. This study aims to determine the incidence and severity of tibia malrotation following MIPO plating of isolated unilateral distal tibia fractures, defined as torsional difference of greater than 10° as compared to the contralateral limb and whether the degree of malrotation affects functional outcomes scores.

**Materials and methods:**

This was a level 2 prospective cohort study. All patients with fractures of the distal tibia who underwent surgical fixation with the exclusion ofpatients with polytrauma, neurovascular injuries or pre-existing disabilities were recruited. Patients underwent MIPO plating followed by a post-operative ComputedTomography (CT) scan of bilateral lower limbs. AOFAS ankle-hindfoot score was recorded at six months and one year follow-up.

**Results:**

A total of 24 patients (28 to 83 years old) were recruited. Nineteen patients obtained CT scans. Nine of the 19 patients (47.3%) had tibia malrotation. The mean tibia malrotation angle was 10.3° (0° - 45°). The average AOFAS scores was 82.4 and 84.3 at 6 months and 1 year follow-up. Degree of CT malrotation was not significantly associated with AOFAS scores at 6 month (spearman rho -0.386) and 1 year (spearman rho -0.343).

**Conclusions:**

Tibia malrotation following MIPO plating of distal tibia fractures is common, with an incidence of 47.3% and an average malrotation angle of 10.3°. The degree of malrotation does not appear to have significant mid-term functional impact on the patient.

## Introduction

Management of distal tibia fractures is challenging forsurgeons and are associated with high rates ofcomplications^1-3^. The proximity of these fractures to the articular surface as well as limited soft tissue cover are main considerations when fixing these fractures. Various osteosynthesis methods have been developed for fixation of these fractures^4^; including intramedullary nailing, open reduction and internal fixation (ORIF), external fixation with or without internal fixation and more recently minimally invasive plate osteosynthesis (MIPO). The principles of MIPO technique include indirect closed reduction, minimal soft tissue and periosteal dissection with a relative stability construct. This allows secondary fracture healing with callus formation and preservation of the bone’s native blood supply and minimises wound related complications. MIPOtechniques have been shown to be superior to conventional osteosynthesis in the tibia and/or fibula, having lower rates of soft tissue related complications^5^. However, one of the challenges of MIPO is difficulty in assessing rotational profile of the limb during fixation.

Tibial malrotation is defined as torsion difference between the affected and unaffected tibia. Given the limited exposure of the surgical incision, the concern that surgeons have with MIPO of the distal tibia is that of difficulty in assessing a limb’s rotational profile. Malrotation followingintramedullary nailing of the tibia has a high incidenceranging from 23% to 41%^6-9^. Comparisons with regards to functional outcomes between distal tibia fractures fixed with an intramedullary nail versus MIPO have shown that either treatment option has similar therapeutic efficacy and functional outcome^10^. While literature shows that such patients have good functional scores at mid to long term follow-up^11^, there is a paucity of data investigating theincidence of malrotation post MIPO.

This study was designed to determine the average tibial malrotation present after MIPO plating and correlate this degree of malrotation with patients’ eventual functional outcome. By doing so, we hope to find out if malrotation is a factor surgeons should be concerned about during MIPO.

## Materials and Methods

This level 2 prospective cohort study was conducted at a level 1 trauma centre. A total of 24 consecutive participants who sustained distal tibia fractures were recruited from Jan 2015 to March 2017. Institutional review board approval was obtained for this study. This study was funded by a grant from AO Trauma Asia Pacific (Project No AOTAP 13-02).

Inclusion criteria were individuals aged 18 and older who sustained unilateral distal tibia fractures (defined as main fracture line within 4cm of ankle joint), underwent MIPO technique of distal tibia fixation, with no previous lower limb fractures, surgery or pre-existing deformity or disability. Exclusion criteria were patients with multiple injuries or those with neurovascular injury or pre-existing disabilities, aged younger than 18 years or with an open physis. Demographic data recorded for each participant at the beginning of the study included age, sex and body mass index (BMI).

All patients underwent MIPO fixation of distal tibia fractures by fellowship trained trauma surgeons. The decision to fix any concomitant fibula fractures and method of fixation was left to the discretion of the treating surgeon. Patients were mobilised immediately post-op with partial weight bearing on the fractured leg. All patients underwent progressive lower limb strengthening with physiotherapy with eventual progression to full weight bearing by three months. Patient’s demographic data and operative information (time to surgery, type of implant) was recorded into a study database. Fracture information was recorded using AO fracture pattern and fracture location, based on the pre-operative radiographs. Post-operative complications including malunion, soft tissue and wound status was recorded.

**Fig 1:**
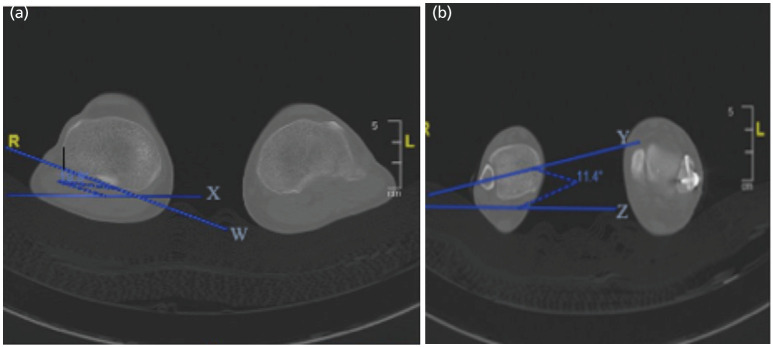
CT Scanogram and technique of measuring rotational profile of the leg. (a) Rotational profile of the leg measured at the knee and (b) above the ankle. (a) A line (line “W”) is drawn tangent to both posterior condyles of the tibial plateau on the CT image just proximal to the fibular head. This angle is measured against the horizontal, labelled line “X”. (b) A distal reference line (line "Y") is drawn through the distal tibia that passes through the centre of the fibula and tibia on a slice just above the distal tibial plafond. This angle is measured against the horizontal, labelled line “Z”. The difference in angles was used to calculate the degree of tibiofibular torsion. The extent of malrotation was assessed as the difference in values between the injured and uninjured limb.

Radiographic assessment of malrotation was done by obtaining post-operative CT images of both lower limbs within the same hospital visit (less than two weeks post-operatively). To accurately quantify the degree of tibial malrotation, we utilized a standard technique similar to those previously described in the literature^8,12,13^. The patient was placed supine, with legs secured to minimize movement during scanning. Proximal and distal transverse axes are determined with CT scanning (Fig. 1). The proximal reference line is determined by drawing a line tangent to the posterior condyles of the tibial plateau on the CT image just proximal to the fibular head. The distal reference line is the transverse axis through the distal tibia that passes through the centre of the fibula and tibia on a slice just above the distal tibial plafond. Tibiofibular torsion is defined as the angle between the two axes. The contralateral limb was used as a control to the affected limb, and the difference between the two limbs was recorded. From references in previous literature^14^, we defined malrotation as a rotational difference of greater than 10° compared to the normal limb.

In addition to CT scans, plain radiographs were done for the patient pre and post-operatively. Pre-operative films were used as initial assessment of the injury pattern (by AO classification), while post-operative films at regular follow-up intervals were used to assess angular reduction and union. All scans were read by two study investigators (orthopaedic surgeons) who were blinded to the patient’s identities, and an average of the data values for each patient was recorded.

Clinical assessment of malrotation was performed by comparing the patient’s prone thigh foot angle by an orthopaedic surgeon. This was done by lying the patient prone, flexing the knee 90° and measuring the angle subtended between the long axis of the thigh and the axis of the foot (line drawn between heel and second toe) with a goniometer.

The AOFAS (American Orthopaedic Foot and Ankle Society) hindfoot score was administered to patients at six months and one year follow-up. This validated clinical rating system combines patient reported subjective scores of pains and function with objective scores based on the surgeon’s physical examination (assessing sagittal motion, hindfoot motion, ankle stability and alignment)^15^.

Statistical analysis was analysed using Stata 13 [StataCorp, College Station, TX]. Categorical variables were presented as numbers and percentage while continuous variable was presented as mean ± SD (standard deviation). Spearman’s rho coefficient was used to measure the strength of associations between the different variables based on normality check results, with a significance level of <0.05 being used. The conformity of continuous variables to normal distribution was evaluated using visual (scatter plots) methods. Unpaired t test was used for the comparison of the mean of data sets that were normally distributed. Mann-Whitney U test was used for comparison of non-parametric data.

## Results

**Fig 2:**
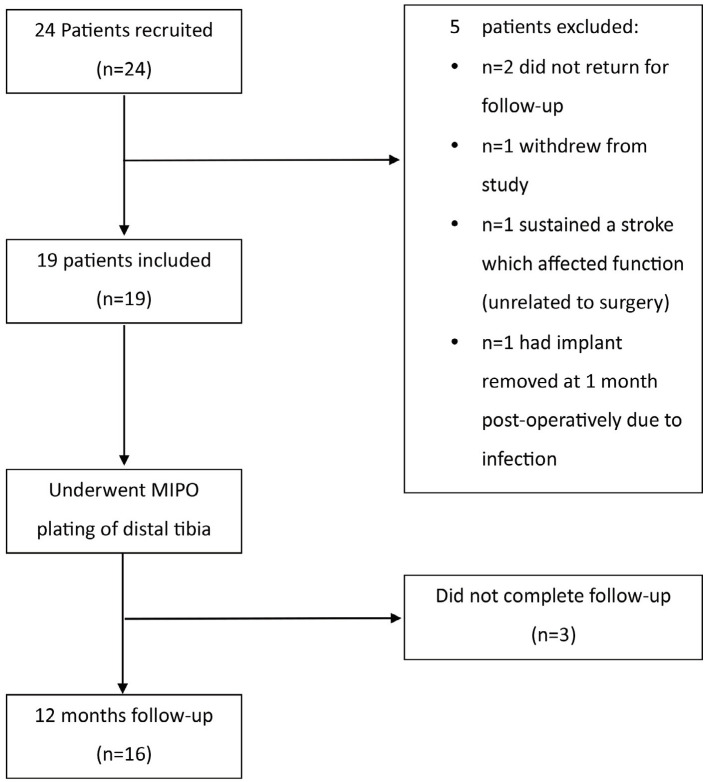
Patient recruitment flowchart.

Of the 24 eligible participants, 19 participants were included in the study, with 16 patients completing follow-up at 12 months. A flowchart showing our patient recruitment is shown in Fig. 2. There were 11 males (57.9%) and 8 females (42.1%). The mean age was 54.3, ranging from 35 to 77 years old. The mean body mass index (BMI) of 26.2 ± 3.50. The demographic information of our study population is summarised in Table I.

**Table I:** Population demographics.

		(n=19)
Age	Mean ± SD	54.3 ± 16.7
Sex, n (%)	Male	11 (57.9)
	Female	8 (42.1)
BMI	Mean ± SD	26.2 ± 3.5
AO classification	A1	8
	A2	3
	A3	7
	C2	1
Concomitant Fibula fracture, n (%)		17 (89.5%)
Fibula fractures fixed, n (%)		15/17 (88.3%)

Based on the AO Classification of distal tibia fractures, 8 patients had type A1 fractures, 3 had type A2, 7 had type A3 and 1 had a type C2 fracture (Table I). Seventeen patients had concomitant fibula fractures out of which 15 underwent plate fixation of the fibula (5 MIPO, 10 conventional).

The mean CT malrotation angle in our study population was 10.3° ± 2.1°. We used absolute values for malrotation regardless of either internal or external rotation or the limb, as the degree of malrotation was dependent on native rotation profile of the normal leg. A total of 47.3% (9/19) of patients had >10º of difference in tibial rotation post-operatively. Majority of patients (16/19) had external rotation of the lower limb. The mean AOFAS scores at 6 months post-surgery was 82.4 ± 12.0. Mean AOFAS scores at 12 months was 84.3 ± 11.3. Table II details the outcomes of our study population.

**Table II:** Patient outcomes.

	Sex	Age	AO	Concomitant	Fibular	MIPO	Order	Rotation (Degrees)					AOFAS Score		Complications
				Fibula Fracture	Fixation	Fibula		Normal Leg	Fracture Leg	CT Malrotation	Rotational deformity	Clinical Malrotation	6m	12m	
1	M	63	A3	Yes	Yes	No	Fibula First	-11	-8	3	ER	10	90	90	Superficial wound infection
2	M	56	A3	Yes	Yes	Yes	Fibula First	32	19	13	ER	15	64	72	Non-union
3	M	40	C2	Yes	Yes	No	Fibula First	32	22	10	IR	5	81	82	Nil
4	F	35	A1	Yes	Yes	No	Fibula First	27	44	17	ER	17	88	95	Nil
5	M	52	A3	Yes	Yes	Yes	Fibula First	46	50	4	IR	4	95	100	Superficial wound infection
6	M	83	A3	Yes	Yes	No	Fibula First	43	48	5	ER	10	94	83	Deep Wound infection
7	M	60	A2	Yes	Yes	No	Fibula First	45	46	1	ER	4	85	90	Nil
8	F	57	A1	Yes	Yes	No	Fibula First	34	22	12	ER	1	84	81	Nil
9	M	77	A3	Yes	Yes	No	Fibula First	7	52	45	ER	20	66	66	Nil
10	M	30	A1	Yes	Yes	No	Fibula First	28	28	0	NA	0	87	87	Nil
11	M	50	A1	Yes	Yes	Yes	Tibia first	37	50	13	ER	8	87	90	Nil
12	F	28	A1	Yes	Yes	No	Fibula first	25	29	4	ER	23	95	95	Nil
13	F	55	A1	No	NA	NA	NA	23	33	10	ER	10	75	84	Superficial wound infection
14	F	76	A2	No	NA	NA	NA	22	37	15	ER	15	89	90	Nil
15	F	63	A3	Yes	No	NA	NA	35	37	2	NA	0	86	88	Nil
16	M	36	A3	Yes	Yes	No	Tibia first	37	22	15	IR	5	53	56	Superficial wound infection, non-union
17	M	48	A2	Yes	No	No	NA	33	45	12	ER	Lost to follow-up			Deep Wound infection
18	F	69	A1	Yes	Yes	Yes	Tibia first	26	27	1	ER				Deep Wound infection
19	F	54	A1	Yes	Yes	Yes	Tibia first	39	53	14	ER				Nil

Key: External Rotation (ER/+), Internal Rotation (IR/-)

We further stratified data to look for common factors between patients with malrotation (>10°) and patients without malrotation (<10°). Patients with A1 or A2 type fractures had a smaller degree of malrotation at 9° ± 6.3°, whilst patients with A3 or C type fractures had a greater degree of malrotation at 12.1° ± 14.1°. This result was not statistically significant at p=0.5211.

**Fig 3:**
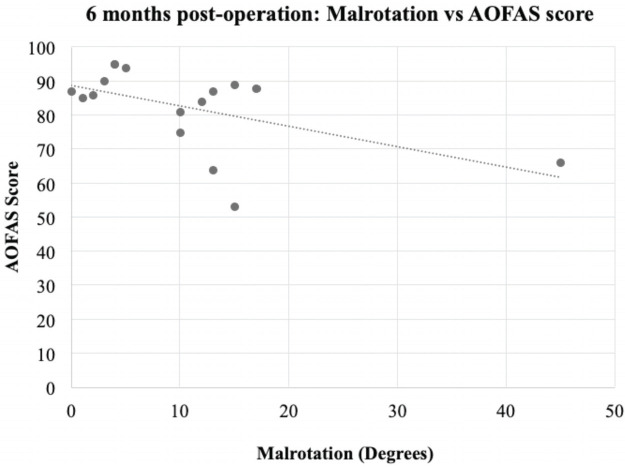
Scatter plot showing Malrotation (degrees) vs AOFAS scores at six months.

**Fig 4:**
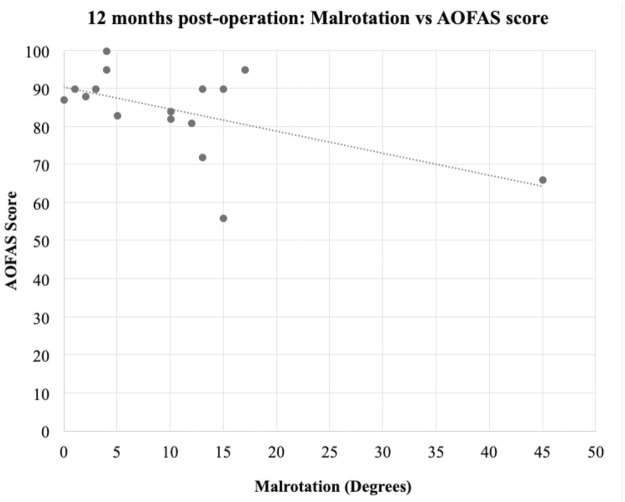
Scatter plot showing Malrotation (degrees) vs AOFAS scores at 12 months.

Spearman’s rho correlation coefficient was used to measure the strength of association between the degree of CT malrotation and AOFAS scores at 6 months and 12 months. Spearman’s rho coefficient was -0.386 (p = 0.14) when compared against AOFAS scores at 6 months. This value was -0.343 (p=0.194) when compared against AOFAS scores at 12 months. While there was a trend toward poorer AOFAS scores with a higher degree of malrotation, this finding was not significant and there was no significant correlation between the degree of malrotation and the AOFAS scores (Fig. 3 and Fig. 4).

A subgroup analysis of patients with concomitant fibula fractures that underwent MIPO, and conventional open reduction and internal fixation was performed. The mean tibial rotational deformity for the MIPO fibula group was 9° ± 4.7° and the conventional group was 11.2° ± 7.8°. This however was not statistically significant (p=0.689).

In our cohort of 19 patients, 3 patients developed complications of deep wound or implant infections requiring repeated wound debridements. The first patient with a deep infection was the oldest patient in our study at 83 years old with a background of poorly controlled eczema presenting with an A3 type open distal tibia fracture. His final AOFAS score at 12 months was fair at 83. The second patient was a 48-year-old male who was a heavy smoker and alcohol drinker, presenting with an A2 type closed distal tibia fracture. The third patient was a 69-year-old lady with poorly controlled diabetes with an A1 closed distal tibia fracture. The last two patients were unfortunately lost to follow-up after their repeat debridements. Four other patients had superficial wound infections that resolved with a course of antibiotics that did not require additional intervention.

Two patients went into non-union and required subsequent revision surgery to address this. The first patient was a 56-year-old male with known diabetes, presenting with an A3 type closed distal tibia fracture that did not show signs of healing and required iliac crest bone grafting. His AOFAS score at 12 months was 72. The second patient was a 36-year-old male who was a heavy smoker with an A3 type open distal tibia and fibula fracture that require iliac crest bone grafting at 6 months. His AOFAS score at 12 months was 56.

**Fig 5:**
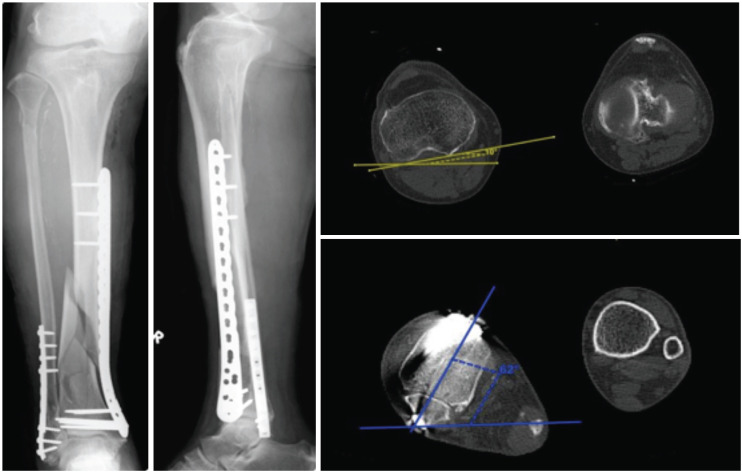
Post-operative radiographs of A3 distal tibia fracture with 45° of malrotation. Post-operative radiographs and Computed tomography scans which demonstrated 52° of external rotation of the distal joint block relative to the posterior tibia condyles. The uninjured limb was in 7° of external rotation with a resultant malrotation of 45° of external rotation.

There was a 77-year-old gentleman with an A3 distal tibia fracture (Fig. 5) with concomitant fibula fracture that had a significant malrotation of 45° post-operatively. On the CT scan, the operated leg was in 52° of external rotation as compared to 7° of external rotation on the non-operated leg. On clinical examination, there was a perceived difference of 20°. Due to extensive comminution of the tibia, an open approach to the fibula was made to aid to restoration of length, alignment and rotation prior to fixation of the tibia. His final AOFAS score was 66 and he was able to ambulate without any aid.

## Discussion

CT scanning of the lower limb is the gold standard for assessment of rotational alignment^8^, and this study shows that the rotational profile of the tibia can be assessed accurately with post-operative CT scans, comparing the operated leg against the normal leg. This avoids the issue of confounders arising from clinical measurements of rotation which also include the thigh, foot and joints as evidenced by the discrepancy between our clinical and radiographical measurements. We defined malrotation as more than 10° of difference between the operated leg and the normal leg, which is similar to other studies^8,14^ in the literature.

Eleven out of 19 patients had a more complex fracture pattern (AO classification A2, A3 or C2) in our study. There was a trend towards a more complex fracture pattern giving rise to a higher degree of malrotation post-operatively, with the highest malrotation angles recorded by the A3 fracture type. Majority of our patients had external rotation of the lower limb, with three patients with internal rotation deformity noted in the A3, C2 fracture pattern group. We attribute this to more complex fracture patterns being more unstable, making it much harder for the surgeon to align the limb to the correct rotational profile intra-operatively as well as having a higher chance of malunion post- operatively. For the A3 fracture with 45° of malrotation post-operatively, on top of the extensive metaphyseal comminution of the distal tibia, the fibula had a proximal fibula neck and a comminuted fracture of the distal fibula. This made it challenging to obtain an ideal reduction of either bone. Due to our small sample size, we were unable to establish if an internal rotation deformity results in poorer functional outcomes.

Intra-operative fluoroscopy has been used to aid in improving rotational alignment of distal tibia fractures, however 47.3% of our study population still had malrotation post-operatively. As a compared to the MIPO technique, malrotation following intramedullary nailing of distal tibia fractures has had varied results. For intramedullary nailing, Prasad *et al*^9^ determined malrotation in 36% of 22 patients, Jafarinejad *et al*^14^ at 30% in 60 patients and Theriault *et al*^16^ at 41% in 70 patients.

Our study incidence of malrotation in MIPO plating (47.3%) is slightly greater to that of intramedullary nailing. This is possibly because limited exposure using MIPO technique makes fracture alignment technically more difficult intra-operatively. Another reason for this could be the higher incidence of periarticular fractures in our patients compared against predominantly mid shaft diaphyseal fractures in the intramedullary studies. Whilst it is easier to judge rotational profile of a tibia with mid shaft fractures by matching proximal and distal cortices, this method cannot be replicated well in periarticular tibia fractures that are comminuted and have poor bone stock.

Our study demonstrated that there are relatively small degrees of malrotation post MIPO plating, with the mean CT malrotation angle averaging 10.3°. Previous studies on reamed intramedullary nailing have shown malrotation angles ranging from 3° to 31°^8,16^. Our results suggest that MIPO plating is comparable to intramedullary nailing, showing an equivalent degree of malrotation post-operatively. Of note is the great variability in the malrotation angles with reamed intramedullary nailing, which may suggest that choosing intramedullary nailing has a steeper learning curve compared with MIPO plating.

We believe one of the key factors helping to reduce the incidence of malrotation would be using the uninjured limb for clinical comparison of the lower limb rotational profile. This can be done prior to draping by assessing the position of the degree of internal or external rotation of the foot in relation to the knee and taking orthogonal radiographs of the uninjured limb as a reference for reduction. Another important factor to minimise malrotation would be accurate placement of the distal tibia plate and screws. While studies have shown no significant difference between different surgical approaches and choice of plating, there is evidence that demonstrates medial plating as having a lower rate of wound related complications^17^. Medial distal tibia plates placed anteriorly over the medial malleolus also shows the least malrotation when compared against middle and posteriorly placed plates^18^. All patients in our study underwent medial plating, and we utilised anatomic implants with a low bend over the distal portion that corresponds with the flare of the distal tibial surface. Distal screws should be placed just above the joint to obtain good subchondral bone purchase.

In the presence of significant comminution or when there is difficulty in obtaining reduction of the distal tibia, reduction and fixation of the fibula may be used as a gauge to determine appropriate length and rotation and hence is thought to improve alignment and rotation. However, there is no clear consensus on the role of fibula fixation in distal tibia fractures with conflicting studies demonstrating improved rotational alignment^19^ and varus/valgus alignment^20^ to having no differences in mechanical complications^21^. In our series, while the mean rotational deformity was less for the MIPO fibula group, this could have been due to surgeons opting to perform MIPO fixation of the fibula if they were confident of obtaining good reduction of the tibia fracture without using the fibula fixation as a guide.

It would be prudent to assess the fracture configuration for both the tibia and fibula and quality of reduction before deciding on whether the fibula required fixation and method of fixation.

We also looked into whether AOFAS scores correlated well with the degree of malrotation measured, with our hypothesis being that the lower the degree of malrotation the better the functional outcome. Our results did show a general trend (Fig. 3, Fig. 4) that people with lower degrees of tibial malrotation have higher AOFAS scores, however our results did not show this relationship to correlate significantly. This leads us to conclude that tibial malrotation is generally well tolerated by our study population and did not have a significant impact on a patients’ functional outcome. This concurs with Cepni *et al’*s study^22^, where despite 51.8% of their patients having malrotation of greater than 10° (mean 14.6°) after MIPO of their distal tibia metaphyseal fracture, there was no significant negative impact on knee and ankle functional scores. Further studies can be done looking into the impact of malrotation of the tibia on such parameters beyond just functional outcomes as well as establish an upper limit for acceptable levels of malrotation.

The high incidence of superficial and deep wound infections in our study despite a MIPO technique reflects the tenuous soft tissue envelope encompassing the distal tibia which may be damaged by the initial injury and further compromised by surgical dissection. This concurs with rates of wound complications of 14.6% by Guo *et al*^23^ and late wound infections following MIPO at 15% by Lau *et al*^24^. This reinforces the need for meticulous soft tissue handling and close monitoring of wounds.

Our study was limited by our small sample size due to limited funding for the post-operative CT scans and short duration of follow-up. Future studies could look into further subgroup analysis differentiating between the different fracture classifications for both the tibia and fibula, the effects of internal and external malrotation and the significance of the order and method of fibula fixation on tibia rotation and functional scores.

## Conclusion

In conclusion, our study found that tibia malrotation following MIPO plating of distal tibia fractures is common, with an average malrotation angle of 10.3°. However, this degree of malrotation does not appear to have any significant mid-term functional impact with patients, showing no significant correlation with the AOFAS scores at 6 months and 12 months.
